# Dementia as a determinant of social and health service use in the last two years of life 1996-2003

**DOI:** 10.1186/1471-2318-11-14

**Published:** 2011-04-06

**Authors:** Leena Forma, Pekka Rissanen, Mari Aaltonen, Jani Raitanen, Marja Jylhä

**Affiliations:** 1 School of Health Sciences, FI-33014 University of Tampere, Finland

## Abstract

**Background:**

Dementia is one of the most common causes of death among old people in Finland and other countries with high life expectancies. Dementing illnesses are the most important disease group behind the need for long-term care and therefore place a considerable burden on the health and social care system. The aim of this study was to assess the effects of dementia and year of death (1998-2003) on health and social service use in the last two years of life among old people.

**Methods:**

The data were derived from multiple national registers in Finland and comprise all those who died in 1998, 2002 or 2003 and 40% of those who died in 1999-2001 at the age of 70 or over (n = 145 944). We studied the use of hospitals, long-term care and home care in the last two years of life. Statistics were performed using binary logistic regression analyses and negative binomial regression analyses, adjusting for age, gender and comorbidity.

**Results:**

The proportion of study participants with a dementia diagnosis was 23.5%. People with dementia diagnosis used long-term care more often (OR 9.30, 95% CI 8.60, 10.06) but hospital (OR 0.33, 95% CI 0.31, 0.35) and home care (OR 0.50, 95% CI 0.46, 0.54) less often than people without dementia. The likelihood of using university hospital and long-term care increased during the eight-year study period, while the number of days spent in university and general hospital among the users decreased. Differences in service use between people with and without dementia decreased during the study period.

**Conclusions:**

Old people with dementia used long-term care to a much greater extent and hospital and home care to a lesser extent than those without dementia. This difference persisted even when controlling for age, gender and comorbidity. It is important that greater attention is paid to ensuring that old people with dementia have equitable access to care.

## Background

Dementia is one of the most common causes of death among old people. In 2007 it was the second most common cause of death among people aged 65 and over in Finland, and in 2009 it accounted for almost half of all deaths in the age group 80 or over [1,2]. In the past two decades the number of deaths caused by dementia has doubled [2], and continues to account for a growing proportion of health and social service use [3].

There is evidence of marked differences in health and social service use between old people with and without dementia. Dementing illnesses are the most important predictor of long-term care among old people [4-8]. In a six-year follow up-study in Finland, 70% of women with dementia and 55% of men with dementia were institutionalized [9]. The research evidence on hospital use is contradictory: some studies indicate that people with dementia are more likely [10] and others that they are less likely [11,12] to be hospitalized than those without the disease. Hospital stays tend to be longer for people with dementia [13,14].

The differences in service use observed between old people with and without dementia are not necessarily due to dementia, but other factors may be at play. It seems that the effect of comorbid conditions varies between different service types. In one study, people with Alzheimer's disease or other dementia used more medical inpatient and outpatient services than those without these diseases because they were physically more ill [15]. Their increased risk of nursing home placement, on the other hand, was not explained by comorbid conditions [16]. However, it is difficult to assess the effect of comorbidity on service use because it is possible that other diseases of dementia sufferers' remain underdiagnosed [17] and thus undertreated.

Studies from different countries have shown that the proportion of old people treated in hospitals in their last year of life has increased over time, but there has been a trend towards shorter hospital stays, for instance in Australia in 1985-1994 [18], in the UK in 1976-1985 [19] and in the USA in 1985-1999 [20].

In Finland, the Ministry of Social Affairs and Health gives preference in its recommendations [21] to home care and sheltered housing over institutional care. The proportion of old people living in sheltered housing increased clearly from 1995 to 2005, while at the same time the proportion of old people in institutional care and home care decreased [3].

In this study, we compared the use of hospital care, long-term care and home care in the last two years of life among people with and without dementia diagnosis from 1996 to 2003. The main focus in earlier studies has been on either acute hospital or long-term care. Our study is population-based, including both people living in their own homes and in long-term care facilities. We hypothesized that old people with dementia use less hospital care and more long-term care in their last two years of life than people without dementia. We also hypothesized that service use among people with and without dementia has changed in line with Ministry of Social Affairs and Health recommendations [21]. The research was conducted as part of the project entitled "Costs of Care Towards the End of Life" (COCTEL). Our research questions were as follows:

1. How does health and social service use in the last two years of life differ between old people with and without dementia?

2. How did health and social service use in the last two years of life among old people with and without dementia change between the years from 1996 to 2003?

To answer these questions we analysed the proportion of service users and the number of days in care among those who used services.

## Methods

### Sample

The sample was drawn from the Causes of Death Register (Statistics Finland). All individuals in the study population were resident in Finland and had died at the age of 70 or over in 1998-2003. The sample consisted of:

1. all those who died at the age of 70 or over in 1998

2. those who belonged to a 40% random sample and died between 1999 and 2001 at the age of 70 or over and

3. all those who died at the age of 70 or over in 2002 or 2003.

For technical reasons it was not possible to include in the sample all deaths for the years 1999-2001. The random sample, representative of the underlying study population [22], was drawn from the Central Population Register of the total Finnish population aged 65 or over, alive on 31 December 1997.

Service use was examined for two years before death (i.e. 730 or 731 days before the day of death). Thus the data include decedents for six years and service use for eight years (since 1996).

### Data sources

The data on health and social service use were derived from the following national registers: Care Register for Health Care, Care Register for Social Welfare and Home Care Census (National Institute for Health and Welfare, THL). The information from these registers was linked using unique personal identification number. A more detailed description of data collection has been given earlier [22]. Days in care were calculated for each individual on the basis of dates of admission to and discharge from care.

Permission to access the register data was obtained from each register controller. The data are not publicly available. The research plan was approved by the Pirkanmaa hospital district ethics committee.

### Services

The services analysed were (1) hospital inpatient care (2) long-term care and (3) regular home care (at least once a week). Hospital use was analysed overall and separately for three types of hospitals representing different levels of care: university hospital, general hospital (central, district and private) and inpatient ward of health centre if the length of stay (LOS) was less than 90 days. Long-term care included residential home, sheltered housing with 24-hour assistance and inpatient ward of health centre (if LOS ≥90 days). Home care included both home nursing and home help. Two outcome measures were used, i.e. (1) any use of individual services during the follow-up, and (2) total number of days in care over potential multiple visits during the follow-up.

### Dementia diagnosis

The dementia diagnoses were identified from the Causes of Death Register, Care Register for Health Care, Care Register for Social Welfare and Home Care Census. A person was categorized as suffering from dementia if in any of the registers they had an ICD-10 code for dementia (F00-F03) or Alzheimer's disease (G30). All aetiologies of dementia were thus included. In addition to the ICD-10 codes, dementia was identified on the basis of class 25 for dementia in a separate 54-grade cause of death classification [23]. We included contributing, immediate, intermediate, and underlying causes of death, and both main and secondary diagnoses in Care Registers.

### Comorbidity

To take into account comorbidity, we identified ten major diagnoses or diagnostic groups from the Causes of Death Register and the Care Registers. These diagnoses were cancer (ICD10-codes C00-C97), diabetes (E10-E14), psychosis, depressive symptoms or other mental health disorders (F04-F99), Parkinson's disease or other neurological diseases (G00-G99 excluding G30, Alzheimer's disease, which is included in the dementia category), chronic asthma and COPD or other respiratory diseases (J00-J99), arthritis or osteoarthritis (M05-M06, M15-M19), hip fracture (S72), stroke (I60-I69), ischemic and other heart diseases excluding rheumatic and alcoholic heart diseases (I20-I25, I30-I425, I427-I52), and other diseases of the circulatory system (I00-I15, I26-I28, I70-I99). From these diagnostic groups we created (1) individual dummy variables for each of the 10 diagnostic categories and (2) a comorbidity variable, indicating the number of other diagnoses except for dementia.

### Analyses

**Figure 1:**
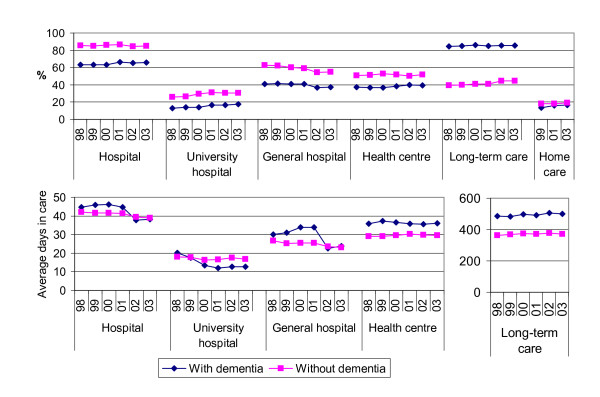
**Proportions of service users and average number of days in care among those who used services in their last two years of life according to year of death**. Adjusted for age and gender.

Comparisons of dichotomous variables were based on chi-square tests, for comparisons of continuous variables we used independent samples t-tests and one-way analysis of variance. The distribution of number of days in care was skewed, and therefore Mann-Whitney U-tests were used to analyse differences in them. Age and gender distributions were different in people with and without dementia. Therefore, for Figure 1, the proportion of services users and number of days in care were adjusted for the age and gender distribution of the whole sample separately for people with and without dementia and for different years of death.

Binary logistic regression models were used to study the likelihood of using different services. The number of days in care was studied for those who used the services at least once during the study period. Data were not available on the number of home care visits. Since days in care variables only yield positive integer values and therefore follow the count data distribution, negative binomial regression models were employed. The independent variables were age, gender, dementia, year of death, an interaction variable of dementia and year of death (dementia*year of death) and dummies for 10 diagnostic categories. If the coefficient of the interaction variable differed from zero (p < .05), additional analyses were performed separately for different years to examine how the effect of dementia differed between the years.

Descriptive analyses and binary logistic analyses were performed with SPSS (15.0) and negative binomial regression analyses were performed with Stata (8.2).

## Results

### Descriptives

The total number of decedents in 1998-2003 was 145,944, of whom 34,232 (23.5%) had a dementia diagnosis (Table 1). On average, people with dementia were 3.5 years older than people without dementia. The proportion of women was higher among dementia sufferers (69.6%) than among non-sufferers (56.2%).

**Table 1:** Descriptive characteristics of old people with (D+) and without (D-) dementia.

	D+	D-	
N for all years	34 232 (23.5%)	111 712	
N by year of death			
1998	7 408 (21.7%)	26 708	
1999*	3 085 (22.2%)	10 811	
2000*	3 124 (22.6%)	10 725	
2001*	3 178 (23.2%)	10 539	
2002	8 700 (24.3%)	27 121	
2003	8 737 (25.3%)	25 808	
			
	Mean (SD)	Mean (SD)	p (t-test)
Average age	85.0 (6.4)	81.5 (7.0)	<.001
Sum of diagnoses	2.0 (1.20)	2.3 (1.16)	<.001
			
	%	%	p (Chi square -test)
Proportion of women	69.6	56.2	<.001
Diagnoses			
Cancer	12.4	26.9	<.001
Diabetes	13.0	15.1	<.001
Mental	8.2	6.8	<.001
Neurological	11.2	10.1	<.001
Respiratory	51.9	43.1	<.001
Arthritis	4.6	6.1	<.001
Hip fracture	10.0	6.8	<.001
Stroke	20.0	23.5	<.001
Heart diseases	46.5	59.7	<.001
Other circulatory	24.6	31.6	<.001
Proportion of users			
Hospital	64.3	85.9	<.001
University hospital	15.1	29.5	<.001
General hospital	38.3	59.1	<.001
Health centre	38.5	51.4	<.001
Long-term care	87.1	40.3	<.001
Home care	14.5	19.2	<.001
Days in care among the users Mean (median)		Mean (median)	p (M-W U-test)
Hospital	41 (25)	41 (30)	<.001
University hospital	14 (7)	18 (10)	<.001
General hospital	27 (10)	25 (15)	<.001
Health centre	36 (32)	29 (23)	<.001
Long-term care	500 (608)	367 (325)	<.001

N = 145 944.

*The sample includes 40% of decedents in this year.

Among dementia sufferers, 32.4% had Alzheimer's disease, 24.7% vascular dementia, 1.9% dementia related to some other disease and 66.0% unspecified dementia. The proportion with more than one dementia diagnosis was 21.6%. In the whole sample the proportion of people with a dementia diagnosis increased annually during the study period (p < .001). The average age at death of both people with and without dementia also increased (p < .001).

The number of other diagnoses was higher among individuals without dementia than among those with dementia (Table 1). Mental, neurological and respiratory diseases and hip fracture were more common among people with dementia, while other diseases were more common among people without dementia.

### Use of different services

A higher proportion of people with dementia used long-term care during the last two years of life than people without dementia (Table 1). People without dementia used all types of hospitals and were clients of regular home care more often than people with dementia.

Among service users, people without dementia had more hospital days overall and in university hospital than those with dementia (Table 1). The number of days in general hospital, health centres and long-term care was higher among people with dementia than among those without it.

### Annual differences over the study period

The proportion of hospital users increased during the follow-up among people with dementia and remained unchanged among people without dementia (Figure 1). The proportions of those who used university hospital or health centre increased, while the proportion of those who used general hospital decreased. These trends were seen both among people with and without dementia, although the changes were different in magnitude. The use of long-term care increased among people without dementia, but remained unchanged among people with dementia. The use of home care increased among people with dementia but no changes were seen among those without dementia.

Among service users, the mean number of days in hospital overall and in university hospital and general hospital decreased over time both among people with and without dementia (Figure 1), but more so among people with dementia. The mean number of days in health centres remained unchanged. Days in long-term care remained unchanged among people without dementia, but increased slightly among people with dementia.

### Multivariate analyses

In models including all independent variables, people with dementia were clearly more likely (OR 9.30, 95%CI 8.60, 10.06) to use long-term care than those without dementia. On the other hand, their likelihood of using all types of hospitals or home care was lower (Table 2).

**Table 2:** Use of services (0 = no, 1 = yes) during last two years of life.

	Hospital								Long-term care		Home care	
			University hospital		General hospital		Health centre					
	OR	95%CI	OR	95%CI	OR	95%CI	OR	95%CI	OR	95%CI	OR	95%CI
Age	**0.97**	0.97,0.97	**0.96**	0.96,0.96	**0.97**	0.97,0.98	**1.01**	1.01,1.01	**1.09**	1.09,1.09	**1.03**	1.03,1.03
Gender (0 = man, 1 = woman)	**0.81**	0.78,0.83	1.02	1.00,1.05	**0.82**	0.80,0.84	**0.95**	0.93,0.97	**1.50**	1.46,1.54	**1.29**	1.26,1.33
Dementia (0 = no, 1 = yes)	**0.33**	0.31,0.35	**0.48**	0.44,0.52	**0.46**	0.44,0.49	**0.58**	0.55,0.62	**9.30**	8.60,10.06	**0.50**	0.46,0.54
Year of death	**0.98**	0.98,0.99	**1.05**	1.05,1.06	**0.93**	0.92,0.93	1.00	1.00,1.01	**1.05**	1.04,1.05	**0.98**	0.98,0.99
Interaction: Dementia * year of death	**1.04**	1.03,1.06	**1.03**	1.01,1.04	**1.04**	1.02,1.05	**1.02**	1.01,1.03	**0.96**	0.94,0.98	**1.08**	1.06, 1.10
Diagnoses (0 = no, 1 = yes)												
Cancer	**3.64**	3.49, 3.81	**1.82**	1.77, 1.87	**1.98**	1.93, 2.04	**1.72**	1.68, 1.77	**0.82**	0.80, 0.85	**1.09**	1.05, 1.13
Diabetes	**1.37**	1.31, 1.43	**0.95**	0.91, 0.98	**1.27**	1.23, 1.31	**1.15**	1.12, 1.19	**1.41**	1.36, 1.45	**1.42**	1.37, 1.48
Mental other than d	1.02	0.97, 1.08	**0.91**	0.86, 0.95	1.03	0.99, 1.08	1.04	1.00, 1.08	**2.04**	1.94, 2.14	**1.26**	1.20, 1.33
Neurological other than d	**1.28**	1.22, 1.35	**1.14**	1.09, 1.18	**1.24**	1.19, 1.28	1.01	0.98, 1.05	**1.74**	1.67, 1.81	**1.20**	1.15, 1.25
Respiratory	**1.42**	1.38, 1.46	**1.08**	1.06, 1.11	**1.20**	1.18, 1.23	**1.21**	1.18, 1.23	**1.52**	1.48, 1.55	**1.13**	1.10, 1.16
Arthritis	**1.36**	1.27, 1.45	0.98	0.93, 1.03	**1.42**	1.35, 1.49	1.01	0.97, 1.06	**1.46**	1.38, 1.53	**1.36**	1.29, 1.43
Hip fracture	**3.45**	3.22, 3.69	**1.57**	1.50, 1.64	**2.15**	2.06, 2.25	0.99	0.95, 1.03	**1.68**	1.61, 1.76	**1.15**	1.09, 1.21
Stroke	**1.28**	1.24, 1.33	**1.08**	1.04, 1.11	**1.18**	1.15, 1.22	**1.04**	1.01, 1.07	**1.51**	1.47, 1.56	**1.07**	1.04, 1.11
Heart diseases	**1.57**	1.52, 1.62	**1.09**	1.07, 1.12	**1.35**	1.32, 1.38	**1.18**	1.15, 1.20	**0.84**	0.82, 0.86	**1.43**	1.39, 1.47
Other circulatory	**1.58**	1.52, 1.63	**1.24**	1.21, 1.28	**1.32**	1.29, 1.36	**1.12**	1.10, 1.15	1.00	0.97, 1.02	**1.19**	1.16, 1.22
Model statistics												
N	145 944		145 944		145 944		145 944		145 944		62 158*	
Nagelkerke R^2^	0.166		0.078		0.106		0.035		0.344		0.042	
-2 Log likelihood	126 682		159 658		189 194		198 231		158 708		134 320	

Binary logistic regression models. Statistically significant (p < .05) odds ratios (OR) are in bold face.

*Data on home care include only years 1999, 2001 and 2003.

Most diagnoses increased the likelihood of using different services (Table 2). Cancer and heart diseases increased the likelihood of hospital use, but decreased the likelihood of long-term care use. Diagnoses of mental disorders decreased the likelihood of university hospital use, but had no effect on the use of other hospitals (p > .05). Most diagnoses also increased the number of days in care (Table 3). We also ran the models using the number of other diagnoses instead of diagnosis-dummies, but the main results remained unchanged.

**Table 3:** Days in care during last two years of life among those who used services.

	Hospital								Long-term care	
			University hospital		General hospital		Health centre			
N	117 974		38 123		79 135		70 595		74 797	
	β	p	β	p	β	p	β	p	β	p
Age	**-0.009**	<.001	**-0.036**	<.001	**-0.019**	<.001	**0.009**	<.001	**0.015**	<.001
Gender (0 = man, 1 = woman)	-0.007	0.384	0.024	0.094	**-0.094**	<.001	**0.090**	<.001	**0.155**	<.001
Dementia (0 = no, 1 = yes)	**0.123**	<.001	**0.144**	0.030	**0.209**	<.001	**0.229**	<.001	**0.231**	<.001
Year of death	**-0.017**	<.001	-0.004	0.205	**-0.027**	<.001	**0.003**	0.045	**0.006**	0.001
Interaction: Dementia * Year of death	**-0.075**	0.002	**-0.308**	<.001	-0.086	0.121	**-0.041**	0.003	0.016	0.067
Diagnoses (0 = no, 1 = yes)										
Cancer	**0.283**	<.001	**0.265**	<.001	**0.191**	<.001	**0.124**	<.001	**-0.237**	<.001
Diabetes	**0.094**	<.001	0.021	0.257	**0.055**	0.002	**0.115**	<.001	**-0.028**	<.001
Mental other than d	**0.230**	<.001	**0.215**	<.001	**0.318**	<.001	**0.158**	<.001	0.010	0.236
Neurological other than d	**0.098**	<.001	0.007	0.758	**0.075**	0.002	**0.126**	<.001	**0.019**	0.008
Respiratory	**0.177**	<.001	**0.201**	<.001	**0.175**	<.001	**0.115**	<.001	0.005	0.313
Arthritis	**0.185**	<.001	**0.128**	<.001	**0.207**	<.001	**0.146**	<.001	**-0.113**	<.001
Hip fracture	**0.077**	<.001	0.025	0.302	-0.004	0.861	**0.207**	<.001	**-0.061**	<.001
Stroke	-0.001	0.916	**-0.124**	<.001	**-0.048**	0.009	**0.076**	<.001	**0.041**	<.001
Heart disease	**0.032**	<.001	0.007	0.668	0.015	0.376	0.011	0.076	**-0.133**	<.001
Other circulatory	**0.091**	<.001	**0.083**	<.001	**0.064**	<.001	**0.068**	<.001	**-0.105**	<.001
Model statistics										
Alpha	0.923		0.983		1.145		0.703		0.769	
Log pseudo likelihood	-555016		-145652		-334300		-310208		-523140	

Negative binomial regression models. Statistically significant (p < .05) coefficients (β) are in bold face.

The likelihood of hospital use, general hospital use and home care use decreased during our follow-up (Table 2). The likelihood of university hospital and long-term care use increased, while the use of health centres did not differ between the study years.

We calculated the interaction term (dementia*year of death) to assess whether the effect of dementia on service use changed by year of death. In all services the effect of this interaction was statistically significant, and we ran additional analyses (not shown) separately for those who died in different years. The differences between people with and without dementia in the likelihood of using each of the services diminished during the follow-up from 1998 to 2003.

Among service users, people with dementia had a higher number of days in care in all types of hospitals and in long-term care than people without dementia (Table 3).

The number of days in hospital overall and in general hospital among services users decreased during the follow-up (Table 3). The number of days in health centres and in long-term care increased over time. The number of days in university hospital remained unchanged (p > .05).

The interaction variable of dementia and year of death was not associated (p > 0.05) with number of days in general hospital and long-term care; a similar trend was seen in both people with and without dementia. Dementia increased the number of days in hospital overall and in health centre less among those who died towards the end of the follow-up (analyses not shown). The diagnosis of dementia increased the number of days in university hospital in the early part of the study period, but decreased that number towards the end of it.

People with dementia were less likely to use hospital care and home care than people without dementia. This is likely due, in part, to their more frequent use of long-term services. Therefore, we also analysed hospital and home care use separately among people with and without dementia who used no long-term care during their last two years of life (analyses not shown). In this sub-sample we found that the use of university and general hospital was less common among people with dementia than among those without dementia, but the use of health centre and home care was more common among those with dementia.

## Discussion

Our aim was to compare the use of health and social services among people with and without a dementia diagnosis during their last two years of life in 1998-2003. We found that people with dementia were more likely to use long-term care but less likely to use hospital care and home care than people without dementia when age, gender, year of death and comorbidity were adjusted for. This was consistent with our hypothesis. Among service users, dementia sufferers spent more days in general hospital, health centre and long-term care than non-sufferers, but fewer days in university hospital.

Although the results describe the Finnish health and social care system and there may be differences between countries, they are broadly consistent with earlier findings from both Finland and elsewhere. It has been reported that dementia is a strong predictor of the use of long-term care e.g. [9,24] but the evidence on the effect of dementia on the use of hospital care is inconclusive. Studies that do not take account of the proximity of death have reported that dementia increases the use of hospital care [10,25,26]. However studies focusing on service use among people in their last years of life have found that dementia decreases hospital use [11,12]. This is supported by the results of the present study. Old people who are in long-term care are less likely to use hospital care, despite their comorbidity, especially those with dementia [27].

We started from the hypothesis that care practices and by the same token service use had changed during our study period from 1996 to 2003. In the case of hospital use the changes were dependent on the type of hospital: the probability of hospital use overall and general hospital use decreased, but the probability of university hospital use increased. In general there was a tendency towards shorter hospital stays, which has been a common trend in other countries over a longer time period [18-20]. Stays were shorter, particularly among people with dementia. Differences in service use between people with and without dementia decreased during the eight-year study period. The changes that were seen over time in service use among both groups may be due in part to organizational changes, or even to changes in the classification of hospitals. However, it is unlikely that such changes will have affected the differences between people with and without dementia.

The use of institutional long-term care increased during the study period. This is at sharp variance with current policy recommendations [21]. We analysed all types of long-term care together, including residential home, sheltered housing with 24-hour assistance and health centres (if length of stay ≥90 days). Therefore, the potential shift from residential care to sheltered housing, which has been reported previously [3] and which is in line with policy recommendations, does not show up in our results.

We found that people without a dementia diagnosis had more other diagnoses than people with dementia. The evidence is conflicting, however: it has been reported both that dementia sufferers have more diagnoses [16,17,28], and the same number of other diagnoses than non-sufferers [29,30]. It has also been suggested that people with Alzheimer's disease are healthier than others [31]. Our data on comorbidity were derived from the Causes of Death Register and the Care Register for Health Care, which includes hospital diagnoses. Because hospital use was more common among people without dementia, their likelihood of having recorded diagnoses will obviously have been higher as well. It is also possible that the smaller number of other diagnoses among people with dementia is due to under-diagnosing [17]. Therefore, our comorbidity variables may underestimate the total level of comorbidity among people with dementia.

We did not have access to information on the time of diagnosis or the severity of dementia, which are important determinants of service use and thus health care costs [24,32,33], and important predictors of nursing home admission [34]. We also lumped Alzheimer's disease and other dementias together, even though there is some indication that service use may differ between them [35].

The proportion of people with a dementia diagnosis in the whole sample increased somewhat during our study period. We do not know whether this was due to improved diagnostic practices or more accurate registration of dementia diagnoses in hospital records, both of which are likely to have happened during our study period, or to decreased mortality among people with dementia. The diagnoses in the registers from which our data were drawn are closely in line with hospital records [36,37]. Still, despite better diagnostics, it is likely that not all cases of dementia in our sample were recorded appropriately in the hospital records [38]. This may be the case especially in the early and mild phase of dementia, and may lead to selection bias towards the most advanced and severe cases. The prevalence of dementia in our sample is closely consistent with the figures for all old people in Finland [39]. However, no data are available on the prevalence of dementia among those living their last years of life.

Our multivariate analyses showed that during their last two years of life, younger old people and men were more likely to use hospital care than older and women, who in turn were more likely to use long-term care. These results confirm earlier findings e.g. [19,40,41]. However, it is not clear whether the effect of age and gender on the use of all services is similar among people with and without dementia. Age has been found to increase the risk for nursing home placement both among people with and without dementia [8], while age and dementia to increase this risk in both genders [42]. It is important that detailed attention is given to possible age and gender differences between old people with and without dementia in service use towards the end of life. Most urgently, however, further research should clarify whether the lower use of hospital care among people with dementia is due to their different needs, or whether it reflects their poorer access to specialized health care.

## Conclusions

In this study we compared service use among old people with and without dementia in the last two years of life in an extensive population sample of people living either in their own homes or in care facilities. We found that people with dementia clearly used more long-term care and less hospital and home care than people without dementia, even though age, gender and comorbidity were controlled for. The results suggest that dementia sufferers' other diseases may remain underdiagnosed and undertreated. It is important to make sure that old people who suffer from dementia have equitable access to care.

## Competing interests

The authors declare that they have no competing interests.

## Authors' contributions

LF: Conception and design of the study, acquisition of the data, analysis and interpretation of the data, drafting the manuscript, PR: Conception and design of the study, acquisition of the data, critical revision of manuscript, MA: Conception and design of the study, critical revision of manuscript, JR: acquisition of the data, analysis and interpretation of the data, critical revision of manuscript, MJ: Conception and design of the study, acquisition of the data, critical revision of manuscript. All authors read and approved the final manuscript.

## Pre-publication history

The pre-publication history for this paper can be accessed here:

http://www.biomedcentral.com/1471-2318/11/14/prepub

## References

[B1] Statistics Finland Causes of Death 2007 (Kuolemansyyt 2007) http://www.stat.fi/til/ksyyt/2007/ksyyt_2007_2008-12-04_tau_002.html

[B2] Statistics Finland Deaths from dementia more than doubled in two decades 2010 Helsinki: Statistics Finland

[B3] Stakes Care and Services for Older People 2005 2007 Helsinki: Stakes

[B4] Viramo P Frey H Erkinjuntti T The health economic implication of dementia (Dementian terveystaloustieteellinen merkitys) Memory disorders and dementia (Muistihäiriöt ja dementia) 2001 Helsinki: Duodecim 37 48

[B5] Luppa M Luck T Weyerer S Konig HH Brahler E Riedel-Heller SG Prediction of institutionalization in the elderly. A systematic review Age Ageing 2010 39 31 38 10.1093/ageing/afp202 19934075

[B6] Aguero-Torres H von Strauss E Viitanen M Winblad B Fratiglioni L Institutionalization in the elderly: the role of chronic diseases and dementia. Cross-sectional and longitudinal data from a population-based study J Clin Epidemiol 2001 54 795 801 10.1016/S0895-4356(00)00371-1 11470388

[B7] Bharucha AJ Pandav R Shen C Dodge HH Ganguli M Predictors of nursing facility admission: a 12-year epidemiological study in the United States J Am Geriatr Soc 2004 52 434 439 10.1111/j.1532-5415.2004.52118.x 14962161

[B8] Andel R Hyer K Slack A Risk factors for nursing home placement in older adults with and without dementia J Aging Health 2007 19 213 228 10.1177/0898264307299359 17413132

[B9] Nihtila EK Martikainen PT Koskinen SV Reunanen AR Noro AM Hakkinen UT Chronic conditions and the risk of long-term institutionalization among older people Eur J Public Health 2008 18 77 84 10.1093/eurpub/ckm025 17566001

[B10] Bynum JP Rabins PV Weller W Niefeld M Anderson GF Wu AW The relationship between a dementia diagnosis, chronic illness, medicare expenditures, and hospital use J Am Geriatr Soc 2004 52 187 194 10.1111/j.1532-5415.2004.52054.x 14728626

[B11] McCormick WC Hardy J Kukull WA Bowen JD Teri L Zitzer S Larson EB Healthcare utilization and costs in managed care patients with Alzheimer's disease during the last few years of life J Am Geriatr Soc 2001 49 1156 1160 10.1046/j.1532-5415.2001.49231.x 11559373

[B12] Rosenwax L McNamara B Zilkens R A population-based retrospective cohort study comparing care for Western Australians with and without Alzheimer's disease in the last year of life Health Soc Care Community 2009 17 36 44 10.1111/j.1365-2524.2008.00795.x 18564194

[B13] Guijarro R San Roman CM Gomez-Huelgas R Villalobos A Martin M Guil M Martinez-Gonzalez MA Toldeo JB Impact of dementia on hospitalization Neuroepidemiology 2010 35 101 108 10.1159/000311032 20551696

[B14] Lyketsos CG Sheppard JM Rabins PV Dementia in elderly persons in a general hospital Am J Psychiatry 2000 157 704707 10.1176/appi.ajp.157.5.704 10784461

[B15] Eaker ED Mickel SF Chyou PH Mueller-Rizner NJ Slusser JP Alzheimer's disease or other dementia and medical care utilization Ann Epidemiol 2002 12 39 45 10.1016/S1047-2797(01)00244-7 11750239

[B16] Eaker ED Vierkant RA Mickel SF Predictors of nursing home admission and/or death in incident Alzheimer's disease and other dementia cases compared to controls: a population-based study J Clin Epidemiol 2002 55 462 468 10.1016/S0895-4356(01)00498-X 12007549

[B17] Lopponen MK Isoaho RE Raiha IJ Vahlberg TJ Loikas SM Takala TI Puolijoki H Irjala KM Kivelä SL Undiagnosed diseases in patients with dementia--a potential target group for intervention Dement Geriatr Cogn Disord 2004 18 321 329 10.1159/000080126 15305110

[B18] Brameld KJ Holman CD Bass AJ Codde JP Rouse IL Hospitalisation of the elderly during the last year of life: an application of record linkage in Western Australia 1985-1994 J Epidemiol Community Health 1998 52 740 744 10.1136/jech.52.11.740 10396507 PMC1756640

[B19] Henderson J Goldacre MJ Griffith M Hospital care for the elderly in the final year of life: a population based study BMJ 1990 301 17 19 10.1136/bmj.301.6742.17 2383701 PMC1663342

[B20] Barnato AE McClellan MB Kagay CR Garber AM Trends in inpatient treatment intensity among Medicare beneficiaries at the end of life Health Serv Res 2004 39 363 375 10.1111/j.1475-6773.2004.00232.x 15032959 PMC1361012

[B21] The Ministry of Social Affairs and Health, The Association of Finnish Local and Regional Authorities National Framework for High-Quality Services for Older People 2008 (Ikäihmisten palvelujen laatusuositus) Helsinki: Ministry of Social Affairs and Health publications

[B22] Forma L Rissanen P Noro A Raitanen J Jylhä M Health and social service use among old people in the last 2 years of life Eur J Ageing 2007 4 145 154 10.1007/s10433-007-0054-4 PMC5546275 28794784

[B23] Statistics Finland 54-grade cause of death classification (54-luokkainen kuolemansyyluokitus) http://www.stat.fi/meta/luokitukset/kuolinsyyt/061-1996/index.html

[B24] Luppa M Luck T Brahler E Konig HH Riedel-Heller SG Prediction of institutionalisation in dementia. A systematic review Dement Geriatr Cogn Disord 2008 26 65 78 10.1159/000144027 18617737

[B25] Goebeler S Jylha M Hervonen A Use of hospitals at age 90. A population-based study Arch Gerontol Geriatr 2004 39 93 102 10.1016/j.archger.2004.01.003 15158584

[B26] Caspi E Silverstein NM Porell F Kwan N Physician outpatient contacts and hospitalizations among cognitively impaired elderly Alzheimers Dement 2009 5 30 42 10.1016/j.jalz.2008.05.2493 19118807

[B27] Burton LC German PS Gruber-Baldini AL Hebel JR Zimmerman S Magaziner J Medical care for nursing home residents: differences by dementia status. Epidemiology of Dementia in Nursing Homes Research Group J Am Geriatr Soc 2001 49 142 147 10.1046/j.1532-5415.2001.49034.x 11207867

[B28] Zhao Y Kuo TC Weir S Kramer MS Ash AS Healthcare costs and utilization for Medicare beneficiaries with Alzheimer's BMC Health Serv Res 2008 8 108 10.1186/1472-6963-8-108 18498638 PMC2424046

[B29] Schubert CC Boustani M Callahan CM Perkins AJ Carney CP Fox C Unverzagt F Hui S Hendrie HC Comorbidity profile of dementia patients in primary care: are they sicker? J Am Geriatr Soc 2006 54 104 109 10.1111/j.1532-5415.2005.00543.x 16420205

[B30] Zekry D Herrmann FR Grandjean R Meynet MP Michel JP Gold G Krause KH Demented versus non-demented very old inpatients: the same comorbidities but poorer functional and nutritional status Age Ageing 2008 37 83 89 10.1093/ageing/afm132 17971391

[B31] Wolf-Klein GP Siverstone FA Brod MS Levy A Foley CJ Termotto V Breuer J Are Alzheimer patients healthier? J Am Geriatr Soc 1988 36 219 224 3339230 10.1111/j.1532-5415.1988.tb01804.x

[B32] Andersen CK Lauridsen J Andersen K Kragh-Sorensen P Cost of dementia: impact of disease progression estimated in longitudinal data Scand J Public Health 2003 31 119 125 10.1080/14034940210134059 12745762

[B33] Lamb VL Sloan FA Nathan AS Dementia and Medicare at life's end Health Serv Res 2008 43 714 732 10.1111/j.1475-6773.2007.00787.x 18370975 PMC2442376

[B34] Gaugler JE Yu F Krichbaum K Wyman JF Predictors of nursing home admission for persons with dementia Med Care 2009 47 191 198 10.1097/MLR.0b013e31818457ce 19169120

[B35] Murman DL Chen Q Colucci PM Colenda CC Gelb DJ Liang J Comparison of healthcare utilization and direct costs in three degenerative dementias Am J Geriatr Psychiatry 2002 10 328 336 11994221

[B36] Sund R Nurmi-Luthje I Luthje P Tanninen S Narinen A Keskimaki I Comparing properties of audit data and routinely collected register data in case of performance assessment of hip fracture treatment in Finland Methods Inf Med 2007 46 558 566 17938779 10.1160/me0382

[B37] Keskimäki I Aro S Accuracy of data on diagnoses, procedures and accidents in the Finnish Hospital Discharge Register Int J Health Sciences 1991 2 15 21

[B38] Ostbye T Taylor DH Jr Clipp EC Scoyoc LV Plassman BL Identification of dementia: agreement among national survey data, Medicare claims, and death certificates Health Serv Res 2008 43 313 326 10.1111/j.1475-6773.2007.00748.x 18211532 PMC2323140

[B39] Sulkava R Koskinen S, Aromaa A, Huttunen J, Teperi J Dementia Health in Finland 2005 Vammala: National Public Health Institute KTL, National Research and Development Centre for Welfare and Health STAKES, Ministry of Social Affairs and Health 86 87

[B40] Bird CE Shugarman LR Lynn J Age and gender differences in health care utilization and spending for Medicare beneficiaries in their last years of life J Palliat Med 2002 5 705 712 10.1089/109662102320880525 12572969

[B41] Nihtila E Martikainen P Household income and other socio-economic determinants of long-term institutional care among older adults in Finland Popul Stud (Camb) 2007 61 299 314 10.1080/00324720701524193 17979004

[B42] Luppa M Luck T Weyerer S Konig HH Riedel-Heller SG Gender differences in predictors of nursing home placement in the elderly: a systematic review Int Psychogeriatr 2009 21 1015 1025 10.1017/S1041610209990238 19589192

